# Optimization of intermittent microwave–convective drying using response surface methodology

**DOI:** 10.1002/fsn3.224

**Published:** 2015-05-11

**Authors:** Nahid Aghilinategh, Shahin Rafiee, Soleiman Hosseinpur, Mahmoud Omid, Seyed Saeid Mohtasebi

**Affiliations:** Department of Agricultural Machinery Engineering, Faculty of Agricultural Engineering and Technology, University of TehranKaraj, Iran

**Keywords:** Bulk density, color change, energy consumption, intermittent microwave–convective, rehydration ratiorehydration ratio

## Abstract

In this study, response surface methodology was used for optimization of intermittent microwave–convective air drying (IMWC) parameters with employing desirability function. Optimization factors were air temperature (40–80°C), air velocity (1–2 m/sec), pulse ratio) PR ((2–6), and microwave power (200–600 W) while responses were rehydration ratio, bulk density, total phenol content (TPC), color change, and energy consumption. Minimum color change, bulk density, energy consumption, maximum rehydration ratio, and TPC were assumed as criteria for optimizing drying conditions of apple slices in IMWC. The optimum values of process variables were 1.78 m/sec air velocity, 40°C air temperature, PR 4.48, and 600 W microwave power that characterized by maximum desirability function (0.792) using Design expert 8.0. The air temperature and microwave power had significant effect on total responses, but the role of air velocity can be ignored. Generally, the results indicated that it was possible to obtain a higher desirability value if the microwave power and temperature, respectively, increase and decrease.

## Introduction

Apple is the fourth major horticultural product of human nutrition in the world. It is the pomaceous fruit of the apple tree, Malus domestica of the rose family of Rosaceae (Forsline et al. 2003). About 69 million tons of apples are produced in the world, half of which is produced in China. Apples are rich in terms of antioxidant. These compounds protect the body from free radical losses. Therefore, apples should be maintained for consumption in all seasons. Drying is one of the primary methods used in food preservation. The objective of drying is to reduce the water to a certain level to minimize the microbial waste (Akpinar and Bicer 2005). Other benefits of fruit drying are the long durability, the need for less storage space, and a lighter weight for transport (Ertekin and Yaldiz 2004). Hot air drying method is the earliest and most widely used drying method. More than 85% of industrial dryers are of convective hot air. The disadvantage of these dryers is their high energy consumption and lower quality. Hence, the necessity of finding fast, safe, and controllable drying methods (Kavak Akpinar et al. 2005; Motevali et al. 2011), as the application of microwave energy to food drying might be a good way to overcome the existing problems in conventional drying methods (Wang et al. 2004; Vadivambal and Jayas 2007). The influence of microwave energy inside a material is dependent on the dielectric properties that can alter the temperature distribution in the sample. In a small sample, the cumulative effect of microwave as a function of time, causes overheat at the center of the sample. In such cases, the use of pulsed heating is the order of the day (Gunasekaran and Yang 2007). On the other hand, if not properly applied, the microwaves are known to result low-quality product. As microwave drying is more appropriate to dry low-moisture content food, it is better to combine microwave drying with other drying methods including convective hot air, vacuum, and freeze drying to achieve more uniform, fast, and effective drying without valuable loss in quality (Wang et al. 2004). IMWC air drying has been successfully used for a number of agricultural products such as potato, carrot (Chua and Chou 2005), sage leaves (Esturk 2012), red pepper (Soysal et al. 2009a), and oregano (Soysal et al. 2009b). Based on the results of (Soysal et al. 2009b), the intermittent microwave–convective drying at 35°C with a pulse ratio of 3 at 597.20 W showed considerable savings in drying time and high-quality product with better physical (color and texture) and sensory attributes when compared to convective air drying. In terms of essential oil content and quality, the IMWC at 25°C room temperature with the pulse ratio of 5 was provided as the most suitable drying method for oregano compared to CMWC drying, convective air dying, and shade drying (Soysal et al.2009a). RSM such as fuzzy logic, artificial neural networks, and genetic algorithms are also empirical models which are widely used for modeling of food processing due to the complexity of the reactions and nonhomogeneous structure of food products. RSM defines the effect of the independent variables, alone or in combination; on the process (Senanayake and Shahidi 2002).This approach enables an experimenter to make efficient exploration of a process or system (Madamba 2002). Therefore, RSM has been frequently used in the optimization of food processes (Eren and Kaymak-Ertekin 2007; Corzo et al. 2008; Wani et al. 2008; Changrue et al. 2008).Concerning the proper combinations of pulse ratio (PR), power of microwave, temperature, and velocity for optimum responses in IMWC, limited studies have been performed up to now. Therefore, the overall objective of this study was to optimize the drying conditions of apple slices in IMCW drier and desirability function concept employed as the strategy for the optimization.

## Materials and Methods

### Microwave–convective drying system

**Figure 1:**
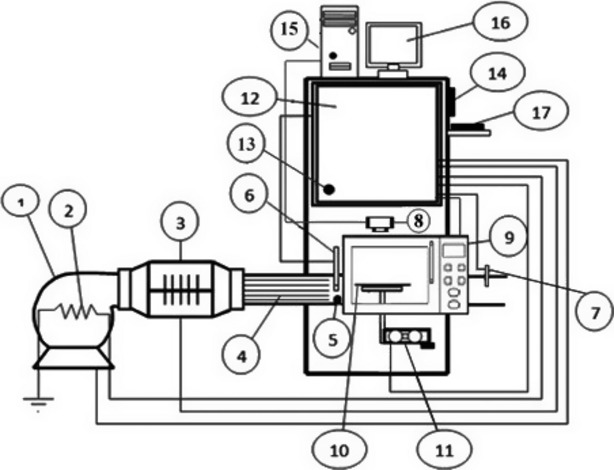
Experimental microwave-hot air dryer. 1. fan, 2. preheating element, 3. heating elements, 4. straightener, 5. medium velocity sensor, 6. relative humidity and temperature sensors, 7. temperature sensor, 8. digital color camera, 9. microwave oven, 10. platform, 11. load cell, 12. control unit, 13. outside temperature sensor, 14. HMI (Human–machine interface), 15. computer, 16. monitor, 17. keyboard.

The schematic diagram of the employed microwave–convective drying system to conduct the experiments is shown in Fig.1.

The dryer consists of a microwave device, centrifugal fan (BEF-25 = 25F4T, 6300 m^3^/h, Damandeh, Tehran, Iran), four electrical heating elements (a 750 W element in the centrifugal fan for preheating the air flow, and three 2000 W elements in the air duct for heating the air flow), a control unit, a single-point load cell, measurement sensors, and a drying chamber with one-layer tray. The body of the dryer was thermally insulated with glass wool. Furthermore, a 2465 MHz domestic microwave oven (Samsung, OM75P, Guangzhou Daeyean Trading Company, China; Samsung Electronics Inc., South Korea) with a 0–1100 W nominal power and cavity dimensions of 300 (width) × 380 (depth) × 260 (height) mm was used. The power supply of the magnetron was controlled by a cycle controller. The microwave used in this study consisted of magnetron, high voltage transformer, diode, and also high-voltage capacitor. The air flow temperature was controlled with an accuracy of ±1°C using a PLC (*Programmable logic controller*), two PT-100 temperature sensors before and after the sample tray. Outside air temperature was measured using a temperature sensor (LM35, NSC, Santa Clara, CA). In addition, relative humidity (RH) of the air flow was determined using a high-temperature RH sensor (EE99-03-FP6AD 802, E+E Elektronik, Engerwitzdorf, Austria). Weight loss and moisture of the samples were measured during the drying by means of a high-precision aluminum single-point load cell (model 1004, Tedea-Huntleigh, Cardiff, UK) with an accuracy of 0.001 g placed under the glass tray (diameter: 314 mm, mass: 1150 g) for a continuous measurement of the mass of the material being dried.

### Sample preparation

Apples (Red delicious variety) used in this study were supplied from a local market and then immediately stored at 6 ± 1°C until the time of the test. Prior to the drying, apples were peeled and cut, perpendicular to the fruit axis in approximately equal slices (6 mm thick). The moisture content was determined by drying the samples at 105°C in the oven for 18 h (Trabelsi and Nelson 2003). The peeled slices had the initial moisture content of about 85% wet basis (w.b.).

### Experimental procedure

The experiments were done at air temperature 40 to 80°C, air velocity at 1 to 2 m/sec, microwave power 200 to 600 W and PR 2 to 6. A PR for IMWC was calculated as PR = (*t*_on_ + *t*_off_) / *t*_on_, where *t*_on_ is magnetron power ON time and *t*_off_ magnetron power OFF time. The on–off timings employed in the IMWC treatments were as following:

30 sec on–30 sec off (PR = 2.0), 20 sec on–40 sec off (PR = 3.0), and 10 sec on–50 sec off (PR = 6.0).

### Bulk density

The bulk density of samples was determined by weighing the samples and then placing them in a container with a determined volume, filled with toluene (Baysal et al. 2003). The volume expansion in the container was recorded at 25°C. The bulk density was determined via the ratio of the samples weight to the volume expanded in cubic meters.

### Rehydration ratio

Rehydration ratio was determined by immersing dried samples in distilled water at specified rehydration temperature (20°C) for 14 h (Lewicki 1998). The water was drained and the samples weighed at every 2-h intervals. The rehydration ratio was defined (eq. 1) as the ratio of weight of rehydrated samples to the dry weight of the sample.

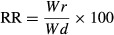
1

### Color change

The amount of color change is usually taken into consideration when comparing the color changes in food drying (Maskan 2000). The sample color was measured before and after drying by a Hunter Lab ColorFlex, A60-1010-615 model colormeter (HunterLab., Reston, VA) at four different points on the apple's surface for all of experiments. The color values were indicated as L* (whiteness/darkness), a* (redness/greenness), and b* (yellowness/blueness). Also, the color change was calculated from the L*, a*, and b* values', using equation 2 and it was used to describe the color change during drying:

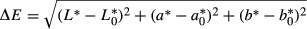
2

where subscript “0” refers to the color reading of fresh apple, and L*, a*, and b* indicate the brightness, redness, and yellowness of dried samples, respectively (Maskan 2000).

### Determination of total phenolic content

A modified method was used to analyze the total phenolic content (TPC) (as gallic acid equivalent (GAE)) using the Folin–Ciocalteu (FC) (Vega-Gálvez et al. 2009). Dried apple (2.00 g), which had been crushed to powder using Waring blender, was mixed with 100-mL cool water at room temperature and constantly swirled with an orbital shaker for 1 h. The extracts were filtered (Whatman filter paper nr 1) in a Buchner funnel. A 0.5-mL aliquot of the apple extract was transferred to a glass tube containing 0.5 mL of FC reagent, vortex-mixed and kept for 5 min. Next, 2 mL of 20% Na_2_CO_3_ was introduced and incubated for 15 min at ambient temperature. After adding 10 mL of ultrapure water, the formed precipitates were removed in 5 min by centrifugation. Finally, absorbance was measured at 725 nm with a spectrophotometer (Spectronic-20 Genesys, IL, USA) and compared to a GA calibration curve. Results were expressed as mg GA/100 g dry matter. All reagents were purchased from Merck chemical company (Merck KGaA, Darmstadt, Germany), and all measurements were carried out in triplicate.

### Energy consumption

Energy consumption, for each drying condition, was estimated based on the drying time involved and energy utilization by the microwave dryer and hot air dryer (eq. 3) (Aghbashlo and Samimi-Akhijahani 2008). It was expressed in terms of MJ of water removed and used as one of the factors in the optimization of process parameters.

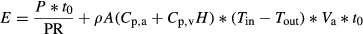
3

where P is power of microwave (W), PR pulse ratio of microwaves, *ρ* bulk density of air (kg/m^3^), *C*_p,a_ heat capacity of air (kJ/kg°C), *C*_p,v_ heat capacity of water vapor (kJ/kg°C), *T*_in_ temperature of inside of drier °C, A area of the sample container (m^2^), *T*_out_ temperature of dryer outside of drier (°C), *V*_a_ air velocity (m/sec), and *t*_0_ time of drying of sample (S).

### Response surface analysis

RSM was used to investigate the main effects of process variables microwave power, temperature, PR, and velocity on the bulk density, rehydration ratio, color change, total phenol content, and consumption energy in this study. Response surface methodology (RSM) is a statistical method that is used to optimize multivariate problems. Also, RSM is effective in determining the relationship between responses and input variables (Kaur et al. 2009; Kumar et al. 2014). RSM was used for statistical analysis in this study by employing Design Expert 8.0 (Stat-Ease Inc., Minneapolis, MN) statistical analysis software. A Box–Behnken design was applied to perform the tests. Three levels of each variable were chosen for the trials, including the central point and two axial points, total of combinations are given in Table1. Experimental data were fitted to a second- or third-order polynomial model. Desirability function (*D*(*x*)) was calculated for optimization of multiple responses (Giri and Prasad 2007; Myers et al. 2009) using eq. 4.

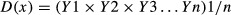
4

**Table 1:** Design matrix processed for the IMWC tests

Run	Velocity (m/sec)		Power (W)		PR		Temperature (°C)	
	Real	Coded	Real	Coded	Real	Coded	Real	Coded
1	1.5	0	600	1	3	0	40	−1
2	1.5	0	200	−1	3	0	80	1
3	1	−1	400	0	3	0	40	−1
4	1	−1	200	−1	3	0	60	0
5	1.5	0	400	0	2	−1	40	−1
6	2	1	400	0	3	0	80	1
7	1	−1	400	0	3	0	80	1
8	1.5	0	600	1	3	0	80	1
9	1.5	0	200	−1	6	1	60	0
10	1.5	0	400	0	3	0	60	0
11	1.5	0	400	0	6	1	40	−1
12	1.5	0	400	0	3	0	60	0
13	2	1	400	0	6	1	60	0
14	1.5	0	400	0	3	0	60	0
15	1.5	0	400	0	3	0	60	0
16	1	−1	600	1	3	0	60	0
17	1.5	0	200	−1	2	−1	60	0
18	2	1	200	−1	3	0	60	0
19	2	1	400	0	2	−1	60	0
20	1	−1	400	0	2	−1	60	0
21	1.5	0	600	1	2	−1	60	0
22	1.5	0	400	0	2	−1	80	1
23	1.5	0	600	1	6	1	60	0
24	1	−1	400	0	6	1	60	0
25	1.5	0	400	0	6	1	80	1
26	1.5	0	200	−1	3	0	40	−1
27	1.5	0	400	0	3	0	60	0
28	2	1	400	0	3	0	40	−1
29	2	1	600	1	3	0	60	0

where, *Y*i (*i* = 1, 2… *n*) are the responses and ‘*n*’ is the total number of responses in the study. The value of ‘*D*’ ranges from zero to one. The ‘*D*’ is a desirability function describing how desirable (well matched) the responses are at a particular level of independent factors (variables). The program of the software search to find the values of variables, which can conclude the maximum value of desirability function (Yadav et al. 2010; Kumar et al. 2014).

#### Model performance evaluation

1. Coefficient of determination (*R*^2^)

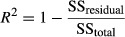

2. Adjusted coefficient of determination (*R*^2^)

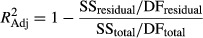

3. Root-mean-square error (RMSE)

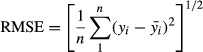

4. Prediction error sum of squares (PRESS)

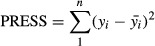

5. Prediction coefficient of determination (*R*^2^)

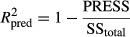

where SS_residual_ is sum of squares of the differences between the predicted and actual values, SS_total_ is sum of squares of the differences between the predicted and average of actual values, yi the ith value of the variable to be predicted, 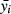 is the predicted value corresponding to *y*_*i*_.

The values of *R*^2^, 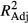, and RMSE are the indicators of how well the regression model fits the observed data. On the other hand, the values of PRESS and 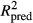 are the indicators of how well the regression model predicts new observations.

## Result and Discussion

### Bulk density

**Figure 2:**
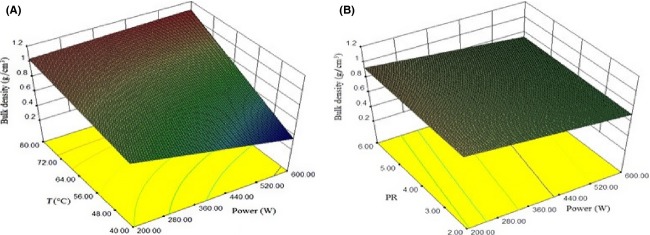
Response surface plot showing the effect of different drying parameters on bulk density (A) power of microwave and temperature (B) PR and power of microwave.

The increase in power of microwave significantly decreased bulk density (*P* < 0.0001). The average values of bulk density of apple slices varied from 0.39 to 1.08 g/cm^−3^. The decreasing trend of the bulk density with an increase in microwave power is in agreement with the findings of previous researchers (Khraisheh et al. 2004; Pimpaporn et al. 2007; Thuwapanichayanan et al. 2011). This might be due to the change in sample texture during drying process, an increase in the microwave power and temperature causes a drop in the apparent and total densities and a slight in the total porosity. Table2 illustrates the fitting results for the models and the best-fitting model is shown in bold and black. The indicators for the selection of the best model that explain change in bulk density was the model with the highest *R*^2^ and 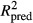, and the lowest PRESS and RMSE values. The variability in bulk density with change in air temperature, PR, and microwave power level is shown in Fig.2. The increasing trend of the bulk density with an increase in PR might be due to the decline in radiation time of the microwave. It is clear from Fig.2A that the bulk density increased faster in higher power because with increasing temperature, positive effect of microwave in increasing porosity decreased.

**Table 2:** Adequacy of the model for bulk density

Model	Statistical index				
	*R* ^2^	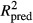	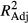	RMSE	PRESS
Linear	0.8771	0.8157	0.8566	0.070	0.17
**2FI**	**0.9443**	**0.8294**	**0.9134**	**0.054**	**0.16**
Quadratic	0.9674	0.8730	0.9349	0.047	0.12
Cubic	0.9761	−1.0766	0.8885	0.061	1.97

R^2^: Coefficient of determination; 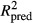: Prediction coefficient of determination; 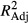: Adjusted coefficient of determination; RMSE: Root- mean- square error; PRESS: Prediction error sum of squares.

### Rehydration ratio

**Figure 3:**
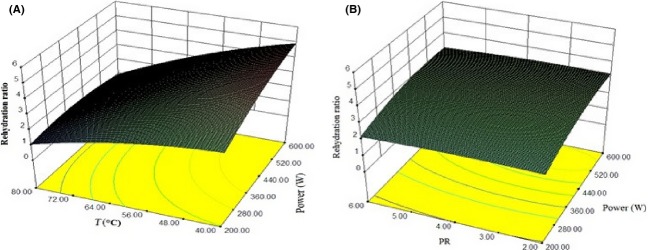
Response surface plot showing the effect of different drying parameters on rehydration ratio (A) power of microwave and temperature (B) PR and power of microwave.

The dried product rehydration characteristic can be used as a quality indicator. Rehydration is a complex process and indicates the physicochemical changes caused by drying (Lewicki 1998). It can be found that microwave power and temperature had significant effect (*P* ≤ 0.01) on rehydration ratio. The coefficients of PR and velocity were not significant (*P* > 0.05). The variability in rehydration ratio with change in air temperature, PR, and microwave power level is shown in Fig.3. According to Sumnu et al. (2005), the high internal pressure produced by microwave drying can cause expansion and puffing of the material and thus reduce the density of the structure. This less dense structure has a higher capacity to absorb water and reconstitute. According to the results presented in Table3, for the rehydration ratio the quadratic model with R2 = 0.9798 was the best.

**Table 3:** Adequacy of the model for rehydration ratio

Model	Statistical index				
	*R* ^2^	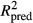	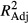	RMSE	PRESS
Linear	0.9021	0.8555	0.8858	0.36	4.65
2FI	0.9375	0.8190	0.9028	0.33	5.82
**Quadratic**	**0.9798**	**0.9003**	**0.9596**	**0.22**	**3.20**
Cubic	0.9927	0.2670	0.9658	0.20	23.57

R^2^: Coefficient of determination; 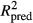: Prediction coefficient of determination; 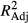: Adjusted coefficient of determination; RMSE: Root- mean- square error; PRESS: Prediction error sum of squares.

### Color change

**Figure 4:**
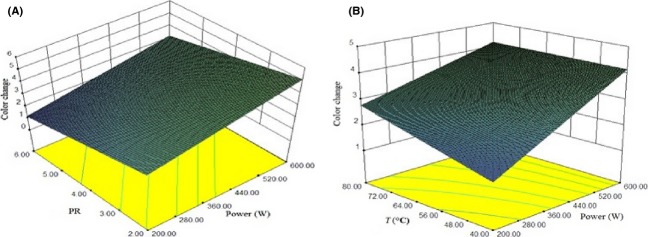
Response surface plot showing effect of different drying parameters on rehydration ratio (A) power of microwave and temperature (B) PR and power of microwave.

The color change in the dried apple slices was characterized in terms of ΔE, which varied from 0.72 to 8.21. It was observed that microwave drying at lowest power in combination with air drying at lowest temperature resulted in lesser color change compared to other treatment combination. This trend may be due to the fundamental decrease in Millard reaction occurred at high air temperature and microwave power level (Kaur and Singh 2014). With increase in temperature and microwave power, there was observed a significant increase in color change (*P* < 0.001). Sharma and Prasad (2006) also observed that dried garlic was darker when higher air temperatures and microwave power levels were used during microwave-assisted drying. Also at higher PR, color change was lower. Also velocity did not have any effect on color (*P* > 0.05). The variability in color change with change in air temperature, PR, and microwave power level is shown in Fig.4.

### Energy consumption

**Figure 5:**
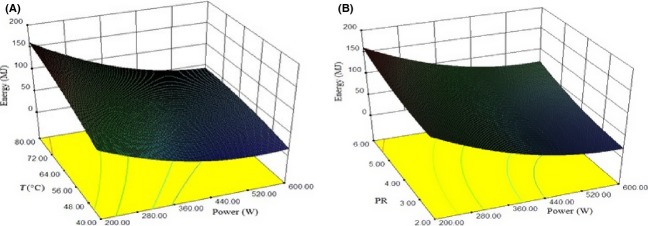
Response surface plot showing effect of different drying parameters on rehydration ratio (A) power of microwave and temperature (B) PR and power of microwave.

Air temperature and microwave power level had significant effect on energy consumption (*P* ≤ 0.05). The effect of process parameters on energy consumption is shown in Fig.5. The regression coefficient of microwave power level was negative. This indicates that energy consumption decreased substantially with increase in microwave power level. This behavior was observed because of considerable decrease in drying time, which reduces the total energy requirements. Similar results were obtained by Sharma and Prasad (2006) for microwave–convective drying of garlic. Multiple linear regression analysis of the experimental data yielded third-order polynomial models with R2 = 0.8199 for predicting energy consumption (Table4).

**Table 4:** Adequacy of the model for energy consumption

Model	Statistical index				
	*R* ^2^	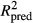	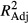	RMSE	PRESS
Linear	0.8399	0.7604	0.8133	19.25	13308.59
2FI	0.8657	0.6158	0.7910	20.36	21342.52
**Quadratic**	**0.8199**	**0.8730**	**0.9396**	**10.94**	**10008.04**
Cubic	0.9953	0.0673	0.9783	6.56	51816.59

R^2^: Coefficient of determination; 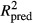: Prediction coefficient of determination; 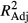: Adjusted coefficient of determination; RMSE: Root- mean- square error; PRESS: Prediction error sum of squares.

### Total phenolic content

**Figure 6:**
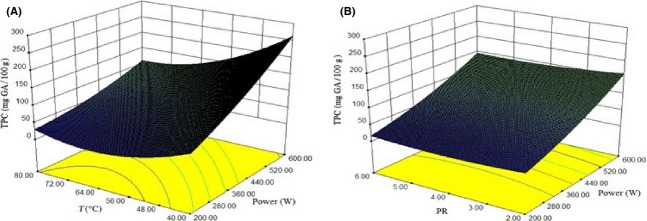
Response surface plot showing effect of different drying parameters on TPC (A) power of microwave and temperature (B) PR and power of microwave.

The TPC significantly increased with the increase in the microwave power (*P *<* *0.01), but it sharply (*P *<* *0.01) fell with the increase in the temperature. However, due to its simplicity and rapidity, microwave treatment could be effective in releasing antioxidant compounds from agricultural byproducts. Long process times ultimately lead to a considerable decrease in nutrient property and antioxidant activity (López et al. 2010). Moreover, exposure to high temperatures disrupts cells and may also result in the release of oxidative and hydrolytic enzymes. These enzymes are capable of oxidizing phenolic compounds. The quadratic model was the best model for the TPC during drying process (Table5). Effect of process parameters on energy consumption is shown in Fig.6.

**Table 5:** Adequacy of the model for TPC

Model	Statistical index				
	*R* ^2^	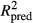	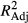	RMSE	PRESS
Linear	0.8236	0.7369	0.7942	27.64	27339.53
2FI	0.8559	0.5151	0.7758	28.85	50388.86
**Quadratic**	**0.9596**	**0.7388**	**0.9193**	**17.31**	**27146.67**
Cubic	0.9912	−1.2780	0.9591	12.32	2.367E+5

R^2^: Coefficient of determination; 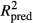: Prediction coefficient of determination; 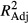: Adjusted coefficient of determination; RMSE: Root- mean- square error; PRESS: Prediction error sum of squares.

### Optimization of IMWC drying of apple

**Figure 7:**
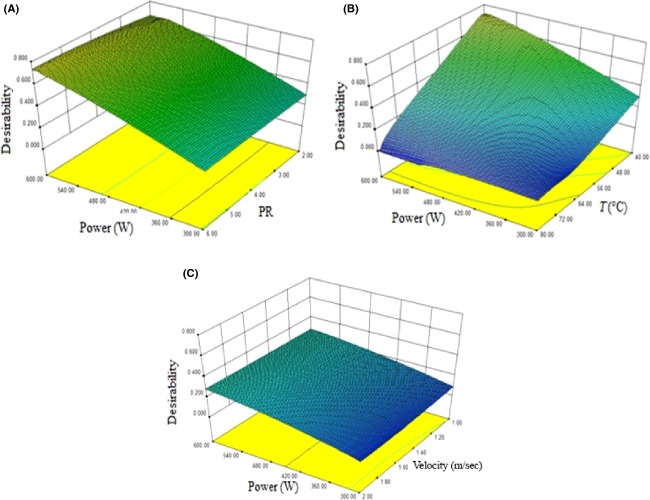
Response surface plot illustrating optimal conditions of desirability as a function of (A) PR and power of microwave (B) power of microwave and temperature (C) power of microwave and velocity.

The desired goals for each variable and response were assigned to adjust the shape of its particular desirability factor (Table6). All factors (variables and responses) were equally weighted. With the application of desirability function method, 52 solutions were obtained for the optimum covering indicators with desirability value of 0.792. Optimum values of process variables with using Design expert 8.0, were: 1.78 m/sec air velocity, 40°C air temperature, PR 4.48, and 600 W microwave power. The values of color change, rehydration ratio, TPC, bulk density, and consumption energy for dried apple slices dried at these drying conditions were predicted as 3.50, 4.88, 256.62, 0.38 g/cm^3^, and 34.12 MJ, respectively. Generally, desirability increases with increasing power of microwave (Fig.7A) and decreases with increasing temperature. It seems that the air velocity and PR had the least effect on desirability function (Fig.7A and C).

**Table 6:** Optimization criteria for different factors and responses

Responses	Goal	Lower limit	Upper limit	Lower weight	Upper weight	Importance
Air velocity (m/sec)	In range	1	2	1	1	3
Power (W)	In range	200	600	1	1	3
PR	In range	2	6	1	1	3
*T* (°C)	In range	40	80	1	1	3
Rehydration ratio	Maximize	1.094	4.77	1	1	3
Color Change (ΔE)	Minimize	0.71801	8.2103	1	1	3
Bulk density (g/m^3^)	Minimize	0.39	1.08	1	1	3
TPC	Maximize	26.123	256.619	1	1	3
Energy (MJ)	Minimize	13.1064	160.164	1	1	3

## Conclusion

The apple was dried from initial moisture content of about 0.85 to 0.1 (w.b.) using IMWC drying method. The experiments were done at air temperature 40 to 80°C, air velocity at 1 to 2 m/sec, microwave power 200 to 600 W, and PR 2 to 6 using a Box–Behnken rotatable design. The drying parameters were optimized based on the quality of dried apple and energy consumed during the drying process. The air temperature and microwave power had significant effect on total responses, but the role of air velocity can be ignored. Generally, the results indicated that it was possible to obtain a higher desirability value if the microwave power and temperature increase and decrease, respectively.

## Conflict of Interest

None declared.
